# Health Care Utilization and Expenditures in Persons Receiving Social Assistance in 2012: Evidence From Switzerland

**DOI:** 10.5539/gjhs.v7n4p1

**Published:** 2014-12-16

**Authors:** Oliver Reich, Felix Wolffers, Andri Signorell, Eva Blozik

**Affiliations:** 1 Department of Health Sciences, Helsana Group, Zürich, Switzerland; 2 Social Welfare Office, City of Bern, Switzerland; 3 Department of Primary Medical Care, University Medical Center Hamburg-Eppendorf, Hamburg, Germany

**Keywords:** health care costs, health care usage, social assistance, social welfare, Switzerland

## Abstract

**Introduction:**

Lower socioeconomic position and measures of social and material deprivation are associated with morbidity and mortality. These inequalities in health among groups of various statuses remain one of the main challenges for public health. The aim of the study was to investigate differences in health care use and costs between recipients of social assistance and non-recipients aged 65 years and younger within the Swiss healthcare system.

**Methods:**

We analyzed claims data of 13 492 individuals living in Bern, Switzerland of which 391 received social assistance. For the year 2012, we compared the number of physician visits, hospitalizations, prescribed drugs, and total health care costs as covered by mandatory health insurance. Linear and logistic adjusted regression analyses were made to estimate the effect of receipt of social assistance on health service use and costs.

**Results:**

Multivariate linear regression analysis revealed that health care costs increased on average by 1 666 CHF if individuals received social assistance. Recipients of social assistance had on average 1.2 more ambulatory consultations than non-recipients and got 1.65 more different medications prescribed as compared to non-recipients. The chance for recipients of social assistance to be hospitalized was almost twice that of non-recipients (Odds Ratio 1.96, 95% confidence interval 1.49-2.59).

**Conclusions:**

Recipients of social assistance demonstrate an exceedingly high use of health services. The need for interventions to alleviate the identified inequalities in health and health care needs is obvious.

## 1. Introduction

Socioeconomic differences in morbidity and mortality are found all over Europe (Mackenbach et al., 2008; Stringhini et al., 2012). Lower socioeconomic position and measures of social and material deprivation are associated with greater morbidity including more frequent occurrence of chronic conditions and higher mortality (Marmot et al., 2012; Lampert et al., 2013; Kroll & Lampert, 2013; Charlton et al., 2013) These inequalities in health among groups of various status constitute one of the main challenges for public health (Marmot, 2012; Pons-Vigués et al., 2014).

As a consequence of their poorer health status the lower socioeconomic strata generally use the health care system more often (van Doorslaer et al., 2006). For example, a comparison of OECD countries found that care from general practitioners was distributed equally, but specialist care was biased to patients with higher incomes (van Doorslaer et al., 2006). It is unknown whether deprived persons cannot sufficiently advocate for their own health which finally results in these inequalities in health care utilization.

In international comparisons, Switzerland repeatedly ranked high with respect to health status and life expectancy (United Nations, 2014). However, even in a rich country such as Switzerland, there is a close connection of socioeconomic determinants with health. The actual National Health Report for Switzerland shows that health and illness have a wide distribution across different socioeconomic groups which finally results in a lower mean life expectancy at birth of more deprived groups (Meyer, 2008).

All persons residing in Switzerland are required to purchase basic health insurance on a private market of health insurance which is regulated by federal bodies. In order to protect those with poor health, health insurers are obliged to offer basic insurance to everyone and to charge the same price to every individual irrespective of age or health status. The basic health insurance package includes medical treatment deemed appropriate, medically effective, and cost-effective. However, the insured person pays a part of the cost of healthcare in the form of an annual deductible ranging from CHF 300 to a maximum of CHF 2 500 as chosen by the insured person and a charge of 10% of the costs up to CHF 700 per year. Currently, there are 61 insurance companies providing basic health coverage in Switzerland, and they offer a range of different premiums and types of health plans from which Swiss residents are free to choose.

In Switzerland, any person who is unable to support himself and his or her family members living in the same household sufficiently or in good time from their own resources is entitled to economic assistance by social aid. The purpose of the provided social assistance is mainly to ensure that basic necessities are met, keep people in society and help them find employment. In 2012, the total social assistance expenditures amounted to 3 billion Swiss Francs (CHF), an annual increase of nearly 7% from the year 2003 (Swiss Federal Office of Statistics, 2012). A total of 250 333 individuals received social assistance (3.1% of the total Swiss population), whereby the rates for the cantons vary greatly between 1.1% (Uri) and 7% (Neuchâtel).

Financial protection in health is strongly associated with health and health care as it diminishes vulnerability and reduces inequities in access to care (Ruger, 2012). Social assistance in Switzerland ensures basic health care by bearing premiums and deductibles of mandatory health insurance. In addition, persons on low income can apply for subsidies of up to 100% to basic health insurance premiums on the cantonal (regional) level.

To the best of our knowledge, no empirically validated findings exist on the health utilization as well as health expenditures of social assistance recipients in Switzerland. Quantifying the medical and economic situation of this socioeconomic deprived population is important to address patients’ needs and to optimize resource allocation. Therefore, the aim of the study was to provide a comprehensive and up-dated overview about the Swiss situation of social assistance recipients compared to non-recipients aged 65 years and younger using claims data from basic health insurance.

## 2. Methods

### 2.1 The System of Mandatory Health Insurance in Switzerland

All persons residing in Switzerland are required to purchase basic health insurance on a private market of health insurance which is regulated by federal bodies. In order to protect those with poor health, health insurers are obliged to offer basic insurance to everyone and to charge the same price to every individual irrespective of age or health status. The basic health insurance package includes medical treatment deemed appropriate, medically effective, and cost-effective. However, the insured person pays a part of the cost of healthcare in the form of an annual deductible (called a ‘‘franchise’’) ranging from CHF 300 to a maximum of CHF 2 500 as chosen by the insured person and a charge of 10 % of the costs up to CHF 700 per year. Currently, there are 61 insurance companies providing basic health coverage in Switzerland, and they offer a range of different premiums and types of health plans from which Swiss residents are free to choose. Social assistance in Switzerland ensure basic health care by bearing premiums and deductibles of mandatory (but not supplementary private) health insurance. In addition, persons on low incomes can apply for subsidies of up to 100% to basic health insurance premiums on the cantonal (regional) level.

### 2.1 Data Source and Study Population

This is a retrospective analysis of individual-level health care claims data from the leading health insurance group (Helsana Group) in Switzerland currently covering about 1.2 million individuals with nationwide mandatory health insurance coverage (about 15% of Swiss residents). We performed a cross-sectional study including all persons living in the Swiss capital, the city of Berne, who were 65 years or younger and enrolled in 2012. A total of 13 492 individuals of which 391 received social assistance were finally included in our study. Characteristics of our sample comprised age, gender, health plan coverage (deductible class (i.e. standard deductible of 300 Swiss francs (CHF) or 500, 1 500, 2 000, or 2 500 Swiss francs, managed care option and availability of supplementary private hospital insurance), and presence of chronic condition using pharmaceutical cost groups (PCG). If medical diagnosis information is missing in the available data set, PCGs are established individual markers for selected chronic conditions (Huber et al., 2013). Our data set also included information on health service utilization, prescription of drugs, and its associated costs from outpatient and inpatient health care settings. These data achieve a high level of completeness since the administrative claims data recorded by insurers cover nearly all health care invoices in the mandatory health insurance scheme.

### 2.2 Statistical Analysis

We applied descriptive statistical techniques to provide a general profile of the study population by comparing persons receiving and persons without social assistance. ATC (Anatomical Therapeutic Chemical) classification system codes were used to discriminate the therapeutical classes of medications. Continuous data are presented as mean values with standard deviation. For the case of categorical variables percentage of patients are given. Differences between the two groups were analyzed with the Kruskal-Wallis test for continuous variables and chi-square test for categorical variables. Patterns of health care utilization were examined per individual for the following variables: physician visits (differentiating whether GP or specialist visits), acute hospital days, psychiatric inpatient days and number of drugs prescribed. Total health care costs were defined as the sum of payments covered by mandatory health insurance for outpatient and inpatient care per patient and year. Outpatient costs included payments for office-based physician visits (primary care physicians, specialists), hospital ambulatory visits, paramedical visits, nursing, laboratory tests, prescription drugs and medical devices. Cost from the inpatient setting comprised payments for hospitalization, rehabilitation, nursing home and emergency transport services, including all associated costs during the inpatient stay (medications, laboratory, medical devices, etc.). Physician visit and hospital length of stay were analyzed for persons with at least one visit or hospitalization in 2012.

To quantify the effect of receiving social assistance on health resource use and in order to get an estimate that adjusts for inter-individual differences in socioeconomic and health-related characteristics, we performed multivariate regression analyses. Linear regression analysis was used to investigate the association between total annual health care costs (independent variable) and receipt of social assistance, number of physician consultations, and the number of different ATC codes (dependent variables). Furthermore, we applied logistic regression analysis to assess the association between hospitalizations (dependent variable) with receipt of social assistance (independent variable). Both linear and logistic regression analysis models were furthermore adjusted for age, gender, deductible class, membership in a managed care plan, and availability of supplementary private hospital insurance. A two-sided p-value <0.05 was considered statistically significant. All statistical analyses were performed using R, version 2.14.2 (R Foundation for Statistical Computing, Vienna, Austria).

### 2.3 Ethics

The analysis complied with the Swiss Federal Law on data protection. All data were anonymized and de-identified prior to the performed analysis to protect the privacy of patients, physicians, and hospitals. Because the data were retrospective, pre-existing, and de-identified, this study was exempted from ethics committee approval.

## 3. Results

Table 1 displays the socioeconomic characteristics of the study population. Of 13 492 individuals residing in Bern (and insured with the Helsana Group), 199 men and 192 women (0.3% of the insured persons) received social assistance. Social assistance recipients were about 4 years younger (33.1 ± 17.2 years) compared to non-recipients (37.2 ± 17.0 years).

**Table 1:** Socioeconomic characteristics of members of the study sample, Bern, Switzerland, year 2012

	Total (Helsana members residing in Bern)	Social assistance recipients	Non-recipients	Difference (95% confidence interval)	*p*^a)^
n	13,492	391	13,101		
proportion	1.000	.029	.971		
***Age***					
Mean	37.1	33.1	37.2	4.1 (2.3, 5.8)	***
***Gender (in %)***					
Male	.497	.509	.496	.013 (0.038, 0.63)	***
Female	.503	.491	.504		
***Deductible class (in %)***					
Swiss francs 0 (children)	.162	.248	.159	.089	
Obligatory (300CHF)	.338	.606	.330	.276	
500 CHF	.144	.087	.145	-0.058	
1,000 CHF	.047	.018	.048	-0.030	
1,500 CHF	.080	.010	.083	-0.072	
2,000 CHF	.021	.008	.022	-0.014	
2,500 CHF	.208	.023	.213	-0.190	
***Member in a managed care plan (in %)***					
No	.408	.652	.401	.251 (.204, .299)	***
Yes	.592	.348	.599		
***Supplementary private hospital insurance (in %)***					
No	.902	.990	.899	.091 (.079, .102)	***
Yes	.098	.010	.101		

* *P* < 0.10, ** *P* < 0.05,

*** *P* < 0.01.

a) Significance level: Kruskal-Wallis-test was used to check for significant differences in age, chi-square-test for differences in the categorical variables.

CHF denotes Swiss Francs.

The majority of recipients of social assistance chose the minimum annual deductible of 300 CHF (61% versus 33%). 35% of social assistance recipients as opposed to 60% of non-recipients were member of a managed care plan that trade off lower premiums for reduced choice and more case management. Almost none of the social assistance recipients had supplementary private hospital insurance (Table 1).

Recipients of social assistance were more frequently suffering from, rheumatologic conditions (45 versus 29%), pain (40 versus 22%), acid related disorders (25% versus 15%), psychological disorders (25 versus 15%), and respiratory illness (10% versus 7%). On a low prevalence level, psychoses (0.9 versus 0.2%) and epilepsy (0.5 versus 0.2%) were also more frequent in recipients as compared to non-recipients. Overall, the proportion of recipients who suffered from one or more chronic conditions was 67% as compared to 50% in non-recipients; and recipients had a mean of 3 as compared to 2.4 different chronic problems in non-recipients (Table 2).

**Table 2:** Clinical characteristics of the study sample, Bern, Switzerland, year 2012

*Chronic condition according to PCG*	Total	Social assistance recipients	Non-recipients	Difference (95% confidence interval)	*p*^a)^
n	13,492	391	13,101		
proportion	1.000	.029	.971		
*Acid related disorders (in %)*	.150	.253	.147	.106 (0.062, 0.149)	***
*Bone diseases (in %)*	.006	.010	.006	.004	
*Cancer (in %)*	.009	.013	.008	.004	
*Cardiovascular diseases (in %)*	.124	.146	.124	.022	
*Dementia (in %)*	.008	.005	.008	-0.003	
*Diabetes mellitus (in %)*	.027	.036	.026	.010	
*Epilepsy (in %)*	.021	.051	.020	.031 (0.009, 0.053)	***
*Glaucoma (in %)*	.009	.008	.009	-0.001	
*Gout, Hyperuricaemia (in %)*	.006	.010	.005	.005	
*HIV (in %)*	.004	.005	.004	.001	
*Hyperlipidaemia (in %)*	.054	.064	.054	.010	
*Intestinal inflammatory diseases (in %)*	.004	.008	.004	.004	
*Iron deficiency, anaemia (in %)*	.036	.051	.035	.016	
*Migraines (in %)*	.011	.015	.011	.004	
*Pain (in %)*	.222	.399	.217	.182 (0.133, 0.231)	***
*Parkinson’s disease (in %)*	.002	.005	.002	.003	
*Psychological disorders (in %)*	.137	.251	.133	.118 (0.074, 0.161)	***
*Psychoses (in %)*	.024	.090	.022	.067 (0.039, 0.095)	***
*Respiratory illness (in %)*	.073	.100	.072	.028 (-0.002, 0.058)	*
*Rheumatologic conditions (in %)*	.290	.453	.285	.168 (0.118, 0.218)	***
*Thyroid disorders (in %)*	.030	.015	.030	-0.015	
*Tuberculosis (in %)*	.001	.000	.001	-0.001	
Chronic conditions (mean)^b)^	2.5	3.0	2.4	0.5 (0.3, 0.8)	***
Proportion with values (%)	.506	.668	.501	.167	

PCG denotes Pharmaceutical Cost Groups.

* *P* < 0.10,

** *P* < 0.05,

*** *P* < 0.01.

a) Significance level: Chi-square-test was used for differences in the categorical variables.

b) For persons with at least 1 chronic condition.

Markers of costs and health care utilization differed statistically significantly between recipients of social assistance and non-recipients. Mean annual health care costs in 2012 were almost double as high (5 596 CHF) for recipients than for non-recipients (2 768 CHF). Consistently, outpatient, inpatient and medication cost were also significantly higher in recipients. Comparing those persons with at least one visit, recipients of social assistance visited on average 1.2 times more frequently a GP and 2.1 times more frequently a specialist per year. For those at least once hospitalized in 2012, length of stay was longer in recipients as compared to non-recipients both for acute, i.e. non-psychiatric (1.9 days longer) and psychiatric hospitalizations (1.5 days longer) (Table 3) However, recipients (6.4%) were 7.5 times more likely than non-recipients (0.9%) to have a psychiatric hospitalization in 2012, but only 1.66 times more likely to have an acute, non-psychiatric hospitalization (12.8% versus 7.7%) (Table 3). Psychiatric hospitalizations contribute to about 36% of all inpatient stays in 2012 in recipients, whereas this proportion is 10% in non-recipients.

**Table 3:** Costs and health care utilization characteristics of study individuals receiving social assistance versus non-recipients in Bern, Switzerland, year 2012

	Total	Social assistance recipients	Non-recipients	Difference (95% confidence interval)	*p*^a)^
n	13 492	391	13,101		
proportion	1.000	.029	.971		
Health care costs mean (CHF)	2850.4	5596.1	2768.4	2827.7 (1729.9, 3925.5)	***
Outpatient costs mean (CHF)	2129.6	3527.5	2087.9	1439.7 (817.0, 2062.3)	***
Inpatient costs mean (CHF)	696.6	2,037.7	656.5	1381.1 (641.9, 2120.4)	***
Medication costs mean (CHF)	628.0	1,005.9	616.7	389.2 (74.2, 704.2)	*
GP visits mean (for persons with at least 1 visit)	5.2	6.3	5.1	1.1 (0.3, 2.0)	**
Proportion with values (%)	.470	.596	.466	.130	
Specialist visits mean (for persons with at least 1 visit)	6.7	8.7	6.6	2.0 (0.5, 3.5)	**
Proportion with values (%)	.487	.529	.486	.043	
LOS acute hospital mean (for persons with at least 1 stay)	7.5	9.3	7.4	1.9 (1.34, 2.46)	***
Proportion with values (%)	.089	.128	.077	.051	
LOS psychiatric hospital mean (for persons with at least 1 stay)	69.8	70.8	69.3	1.5 (1.32, 4.32)	***
Proportion with values (%)	.010	.064	.009	.055	
Different ATC-codes mean (for persons with at least 1 drug)	6.8	9.1	6.7	2.4 (1.5, 3.4)	***
Proportion with values (%)	.655	.785	.651	.134	

CHF indicates Swiss francs; GP, general practitioner; LOS, Length of stay; CC, Chronic Condition; ATC, Anatomical Therapeutic Classification.

* *P* < 0.10,

** *P* < 0.05,

*** *P* < 0.01.

a) Significance level: Kruskal-Wallis-test was used to check for significant differences between the groups.

Medication prescriptions were more frequently in recipients: 79% of recipients as compared to 65% of non-recipients received at least one prescription, and recipients had a mean of 9.1 different substance groups (according to ATC codes) as compared to 6.7 in non-recipients (Table 3).

**Figure 1:**
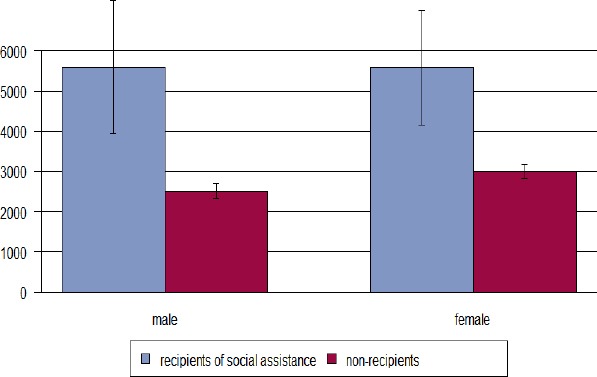
Mean total health care costs (Swiss Francs) and gender for persons receiving social assistance versus non-recipients in Bern, Switzerland, year 2012

**Figure 2:**
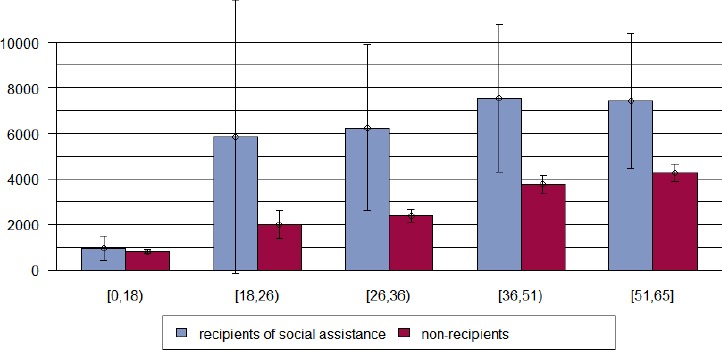
Mean total health care costs (Swiss Francs) and age group for females receiving social assistance versus non-recipients in Bern, Switzerland, year 2012

**Figure 3:**
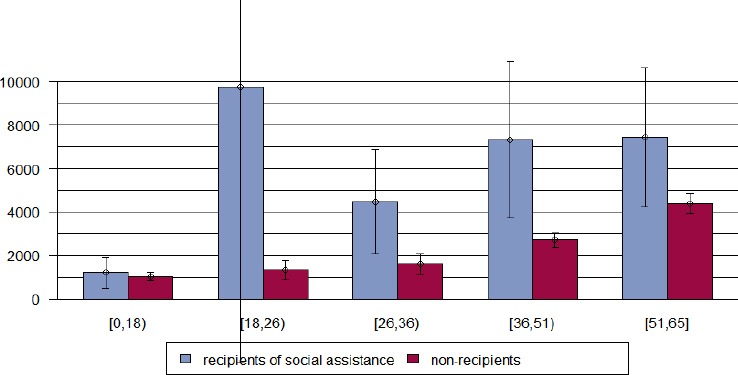
Mean total health care costs (Swiss Francs) and age group for males receiving social assistance versus non-recipients in Bern, Switzerland, year 2012

Figure 1 illustrates that female individuals from Bern who do not benefit from social assistance generate higher costs than men, whereas there is no such gender effect for recipients of social assistance. Stratifying the data according to age class reveals that health care costs increase with age both for recipients and non-recipients –as can be expected – but this effect is more pronounced in non-recipients as they start on a much lower level in younger ages Figures 2 & 3). Males in the youngest age class investigated (19-26 years) had extraordinary expenses that exceeded by far those of the higher ages classes, however, this findings is due to the small sample size in this age class (N=15) so that an outlier of 90 750 CHF massively affected the mean of that subgroup (Figure 3). When excluding this outlier, mean cost were 3 750 CHF.

For quantification of the effect of receipt of social assistance on health resource use controlled for differences in age, gender, and insurance status, we performed multivariate linear regression analyses. According to these analyses, health care costs increased on average by 1 666 CHF if individuals received social assistance. The estimated coefficient was statistically different from zero at the 99% confidence level (Table 4). With respect to ambulatory health service use, recipients of social assistance had on average 1.2 more ambulatory consultations than non-recipients, a highly statistically significant estimate. As for medication usage, recipients of social assistance had 1.65 more different medications prescribed. This estimates, too, were significantly different from zero on the 99% confidence level. In addition, the chance for recipients of social assistance to be hospitalized was almost twice that of non-recipients (Odds Ratio 1.96, 95% confidence interval 1.49-2.59) (Table 4). The complete models derived from linear and logistic regression analysis are available upon request.

**Table 4:** Results of multivariate regression analyses estimating the effect of receipt of social assistance on health care resource use, year 2012

Parameter of health resource use	Effect estimate	Measure of precision	Statistical testing

	Coefficient estimate^a^	Standard error	p-value
Total annual health care cost (CHF)	1665.88	(378.14)	0.000 ***
Number of physician consultations	1.22	(0.45)	0.006 **
Number of different ATC codes	1.65	(0.29)	0.000 ***
hospitalization	Odds ratio^b^	95% confidence interval	p-value
	1.964	1.490-2.588	0.000 ***

* *P* < 0.10,

** *P* < 0.05,

*** *P* < 0.01.

CHF denotes Swiss Francs.

a derived from linear regression analysis, adjusted for age, gender, deductible class, membership in a managed care plan, and availability of supplementary private hospital insurance.

b derived from logistic regression analysis.

## 4. Discussion

This is the first study investigating differences in health resource utilization between recipients and non-recipients of social assistance in Switzerland. It detected considerable inequalities in health care expenditures and usage. Specifically, recipients of social assistance used the health system more frequently than parts of the population that were not in need of social assistance. This resulted in considerable cost for healthcare needs of this deprived population.

Inpatient costs seem to be the main factor for higher total health care costs in recipients of social assistance. Psychiatric services (i.e. hospitalizations) contribute disproportionally to the magnitude of the difference between recipients and non-recipients. As recipients of social assistance were more likely to suffer from neuro-psychiatric problems including pain syndromes these clinical problems seem to be the main drivers of hospitalizations in the deprived population. This assumption is in line with previous research indicating an increased prevalence of mental disorders in deprived populations (Reijneveld & Schene, 1998). Clearly, this is a typical chicken-and-egg situation, and given the cross-sectional nature of our analysis, it is not possible to draw conclusions from our results of whether social deprivation raises, facilitates, or exacerbates, neuropsychiatric conditions, or whether persons with mental disorders are more likely to become dependent on welfare assistance. Most likely, both are true (Thornicroft, 1991). As the increase of length of stay in acute and psychiatric hospitals was not of the same magnitude as the increase in inpatient costs, we hypothesize that the complexity and/or severity of medical problems that lead to hospitalizations in the deprived population is much higher than in those better off.

The higher prevalence of acid related disorders may be associated with medication-induced problems of the gastrointestinal system. For example, the increased prevalence of pain syndromes which are often managed using non-steroidal anti-inflammatory drugs may induce an increased need for antacids in the deprived population. Respiratory illness such as asthma or chronic obstructive pulmonary disease was also more frequent in recipients of social assistance. This finding is in line with previous research showing that social deprivation is associated with risk factors for these conditions, primarily with smoking behavior, but also with exposition to air pollution (Sofianopoulou et al., 2013; Nevill et al., 2014; Jordan et al., 2014).

Based on the present analysis, we have no indication that deprived populations have limited access to healthcare in Switzerland. As opposed to previous studies from other European countries, we did not find a pro-rich distribution for consultations both of primary care physicians and specialist care (van Doorslaer et al., 2006). However, detecting inequalities in health care usage in general is principally in line with data from other European countries (van Doorslaer et al., 2006; Mackenbach et al., 2008; Stringhini et al., 2012; Lampert et al., 2013). In addition, our results generally concur with a previous investigation of long-term unemployed individuals from Bern (Wolffers, 2012). This evaluation detected a remarkable gender difference: health care costs of young males were more than twice as high than those of men from the general population. However, this previous study relied on aggregated data from the association of health insurers, so that it was not possible to control for differences between the examined groups on the individual level. Our study also found a striking difference in health care costs of male recipients of social assistance in the youngest age class of working age, but this finding must be interpreted with utmost caution due to statistical imprecision.

A potential limitation of our study is that the analyses were done for inhabitants of the city of Bern which may limit the generalizability to the total population of recipients of social assistance in Switzerland. The GDP per inhabitant in 2012 in Bern was higher (93 165 CHF) as compared to the mean for Switzerland (74 140 CHF) (City of Bern, 2013a), and the rate of unemployment in Bern in 2012 (2.9%) was slightly smaller than the rate for Switzerland (3.3%) (City of Bern, 2013b). However, given the magnitude of the detected effects it is very unlikely that differences across the social structure that are specific to the city of Bern completely simulated these effects. In addition, we investigated invoicing data which means that inferences to the health status and health needs of the population in need of social assistance can only be made indirectly. Further research is needed to deepen the insight into reasons and motivations for the high level of health service use of the deprived population. Health problems, prevalence of acute and chronic reasons for encounter and the related health care needs need to be explored in more detail. For example, it is unclear whether the increased usage of health services by recipients of social assistance directly translates into worse health or whether a certain proportion of consultations results from inappropriate referral or self-referral due to unmet needs in the social or medical system. In addition, it should be explored if medical problems in the deprived population are more complex and more severe, what factors make the care for those individuals more difficult and whether these are modifiable. The Capability Approach, for example, may be helpful for use in future studies disentangling the link between health status, health service usage and social deprivation as it includes interrelated aspects of health expenditure, such as direct and indirect costs of illness, individual coping strategies used to meet costs, or household consumption patterns (Ruger, 2012).

Negative consequences of inequalities in health care needs between recipients of social assistance and non-recipients concern large parts of the Swiss society. The substantial cost for healthcare needs of recipients of social assistance represent a major burden for the tax-financed system of social assistance in Switzerland, and budgets of cantons and municipalities are severely constrained. As the main part of health care cost, apart from premiums and deductibles, is covered by basic health insurance, the total collective of health insurance premium payers also shoulders a considerable financial burden.

## 5. Conclusion

Recipients of social assistance exhibit an exceedingly high usage of health services which very probably corresponds to a particularly precarious health status. The need for interventions to alleviate the identified inequalities is obvious. This study identified mental health problems as one main targets for interventions. As individuals in this population are likely to have a complex set of socio-medical problem constellations interventions that base on principles of case management may be promising (Björkman & Hansson, 2000; Gask et al., 2012; Lamb et al., 2014). Of course, potential future interventions to ameliorate the health of deprived populations and to optimize the use of health resources should be accompanied by scientific evaluation.
